# Comparative Control of *Phyllotreta striolata*: Growth-Inhibiting Effects of Chemical Insecticides Versus the Green Advantages of a Biopesticide

**DOI:** 10.3390/insects16060552

**Published:** 2025-05-23

**Authors:** Fuyong Lin, Musa Hassan Muhammad, Yufei Mao, Fan Zhao, Zixuan Wang, Yongcong Hong, Pumo Cai, Xiong Guan, Tianpei Huang

**Affiliations:** 1 State Key Laboratory of Agricultural and Forestry Biosecurity & Key Laboratory of Biopesticide and Chemical Biology of Ministry of Education & Biopesticide Research Center, College of Life Sciences, Fujian Agriculture and Forestry University, Fuzhou 350002, China; fuyonglin06@126.com (F.L.); musa.hassan@fud.edu.ng (M.H.M.); maoyufei0109@163.com (Y.M.); 18656905575@163.com (F.Z.); wangzx@fafu.edu.cn (Z.W.); xiongguan@fafu.edu.cn (X.G.); 2 College of Tea and Food Science, Wuyi University, Nanping 354300, China; wyxyhyc@wuyiu.edu.cn (Y.H.); caipumo@wuyiu.edu.cn (P.C.)

**Keywords:** *Phyllotreta striolata*, biopesticides, chemical pesticides, *Brassica chinensis*, integrated pest management

## Abstract

*Phyllotreta striolata* is a major pest of *Brassica chinensis*, with chemical pesticides being the primary method for pest control. However, their negative impact on crop growth and environmental risks have prompted the search for low-toxicity, environmentally friendly alternatives. This study systematically evaluates the control effectiveness of two commonly used chemical pesticides, Zu Jia^®^ and Jie Tiao^®^, along with their impact on the growth characteristics of *B. chinensis* and compares them to the biopesticide *Bacillus thuringiensis* (Bt) strain G033A. The control efficacy was assessed by measuring the insect population reduction rate and plant physiological indicators (weight, plant height, chlorophyll content, and root length). The differences between biopesticide and the two chemical pesticide treatments were analyzed. The results indicated that both Bt strain G033A and the chemical pesticides significantly controlled *P. striolata*. However, chemical pesticides (Zu Jia^®^ and Jie Tiao^®^) significantly reduced the chlorophyll content, plant height, and weight of *B. chinensis* (*p* < 0.05), whereas Bt strain G033A had no significant adverse effects on these plant traits (*p* > 0.05). The colonization characteristics of the Bt strain suggest potential for sustained pest control, while chemical pesticides exhibited rapid pest suppression effects. The findings highlight a novel IPM strategy that integrates the long-lasting efficacy of Bt biopesticides with the rapid action of chemical pesticides, offering a more sustainable and balanced approach to *P. striolata* management in *B. chinensis* cultivation.

## Simple Summary

This study investigates the control effectiveness of two commonly used chemical pesticides and one biopesticide for managing *Phyllotreta striolata* and evaluates their impact on promoting the growth of *Brassica chinensis*. The results showed that the biopesticide significantly reduced pest populations and improved plant growth, including plant height, root length, and weight. Importantly, the biopesticide had no negative impact on chlorophyll content, demonstrating its environmental friendliness. Although chemical pesticides provided faster pest control in the short term, they had some inhibitory effects on plant growth. In contrast, the biopesticide offered a more sustainable and eco-friendly alternative. The findings suggest that combining biopesticides with chemical pesticides may provide a more effective and environmentally responsible solution for managing *P. striolata*.

## 1. Introduction

*Brassica chinensis* belongs to the Brassica genus of Cruciferae, also known as Shanghai cabbage, rapeseed Qingjiang cabbage, etc. *Brassica chinensis* is widely cultivated in the world, especially in East Asia, and it plays an important role in the residents’ diet [1]. It provides people with rich nutrition, including vitamins, minerals, dietary fiber, etc., which helps maintain human health. It also occupies an important position in agricultural production. It is one of the important agricultural products for farmers to plant and sell and has a positive effect on increasing farmers’ income and promoting rural economic development [2,3].

*Brassica chinensis* thrives in autumn and winter, which coincides with periods of high pest and disease incidence. Among these pests, *Phyllotreta striolata* is particularly harmful, causing annual economic losses estimated at USD 300 million [4,5]. As the most common flea beetle species, *P. striolata* poses significant threats to crop production. Current control methods include physical, agricultural, chemical, and biological approaches [6]. Physical control methods, such as light trapping and mulching, can reduce pest populations but are labor-intensive and less effective for hidden or non-phototactic pests [7]. Agricultural practices like weeding and applying plant ash offer temporary relief but are insufficient under high pest pressure [8]. Chemical and biopesticides currently represent the primary strategies for controlling diamondback moth populations. While chemical pesticides offer rapid and effective suppression of pest populations, they also present significant challenges including the development of resistance, environmental contamination, and risks to non-target organisms [9,10,11,12,13]. On the other hand, biopesticides such as azadirachtin, imidacloprid, and Bt agents are characterized by their low toxicity, minimal residual effects, and enhanced environmental safety [14,15,16,17,18]. However, their efficacy in pest control is often slower [19,20]. Given the complementary strengths and limitations of these methods, it is crucial to assess the comparative efficacies of various chemical and biological formulations. Integrated pest management (IPM) strategies that amalgamate the rapid action of chemical agents with the ecological benefits of microbial pesticides (e.g., Bt) offer a promising avenue for the effective and sustainable control of *P. striolata*.

Zu Jia^®^ is a wettable powder formulation containing imidacloprid and abamectin, characterized by good dispersibility and ease of storage but relatively lower penetration ability. In contrast, Jie Tiao^®^ is a microemulsion formulation of the same active ingredients, which offers enhanced permeability, better adhesion to plant surfaces, and improved stability under field conditions. These formulation differences can influence the pesticide’s bioavailability and field efficacy. For biological control, Bt strain G033A produces Cry toxins, a class of insecticidal crystal proteins that specifically target coleopteran pests by disrupting midgut epithelial cells, leading to pest death. The combination of different pesticide types and formulations in this study aimed to explore their relative performance against *P. striolata* and their potential roles in integrated pest management (IPM) strategies.

This study targeted *P. striolata* as a pest of interest, using three treatments: Zu Jia^®^, Jie Tiao^®^, and Bt G033A. Conducted during the winter season, this study employed physiological measurements and pest reduction rates to systematically compare the efficacy of different chemical pesticide formulations, as well as chemical versus biological pesticide treatments. The findings provide scientific guidance for effective *P. striolata* control in winter-grown *B. chinensis*, with significant practical implications for optimizing pest management in these systems.

## 2. Materials and Methods

### 2.1. Experimental Site, Layout, and Cultivation

The experiment was conducted during the winter of 2019 and repeated in 2020 at Guashan Village (25°91.63′ N, 119°26.89′ E), Nantong Town, Minhou County, Fuzhou City, Fujian Province, China. The climate of this region is favorable for the cultivation of Brassica crops, and the soil type is typical of the agricultural soils in eastern Fujian. The average monthly sunshine duration is approximately 100 h (with a sunshine rate of 40%), the total rainfall is about 100 mm, and the volumetric water content of the topsoil is approximately 20–25%. The main type of soil is loam. The temperature ranged from 9 to 21 °C, with predominantly overcast weather. A total of 24 experimental plots, each measuring 10 m², were established for the field trial. The experimental design followed a completely randomized block design, ensuring that the samples in each treatment group were evenly distributed. All crops were managed in strict accordance with the “Organic Product Production Technical Regulations” (DB43/T 1624-2019 [21]), with the application of “Tiandi” compound fertilizer (16-16-16) and urea. No other chemical fertilizers or pesticides were used during the crop management process.

### 2.2. Treatments and Application

Based on weekly scouting for *P. striolata* larvae, the application of treatments began 6 weeks after planting, when the average larval density reached the control threshold of approximately 2 larvae per plant, primarily consisting of first and second instar larvae. Weekly applications were made until the harvest of Shanghai cabbage at week 8. Distilled water was used as the control treatment. For the field application (Table 1), a backpack sprayer was used. Due to the UV sensitivity of the biopesticides used, applications were performed in the evening. Each treatment was replicated six times.

### 2.3. Data Collection

#### 2.3.1. Pest Reduction Rate

Data collection was performed using a five-point sampling method. Four corner points and the center of each plot were marked, and at each point, the number of *P. striolata* adults was recorded on 20 plants. The insect population reduction rate was calculated using the following formula: [(A − B) ÷ A] × 100%, where A is the pest density in the control group and B is the pest density in the treatment group. Data were collected on the 1st, 3rd, 5th, and 7th days after the final application.

#### 2.3.2. Plant Height, Root Length, and Weight

On the 7th day after the final application, one healthy plant was randomly sampled from each plot, with a total of six plants per treatment. Plant height, root length, and plant weight were then measured.

#### 2.3.3. Chlorophyll Content

On the 7th day, one leaf free from lesions and mechanical damage was selected from each plot, with a total of six leaves per treatment. Chlorophyll content was measured using a portable chlorophyll meter (TYS-4N, Beijing Jinkelida Electronic Technology, Beijing, China). Measurements were taken in the central region of each leaf, avoiding the main vein.

#### 2.3.4. Bt Colony Count

On the 7th day after the final application, one healthy leaf free from mechanical damage was randomly collected from each plot, with a total of six leaves per treatment. The central portion of each leaf was excised using sterile scissors and placed into a 10 mL sterile physiological saline solution in a centrifuge tube. The solution was gently shaken to release the bacterial suspension. The suspension was then filtered to remove leaf debris and diluted to 10^−4^. A 100 μL aliquot was spread on LB agar plates containing 20 μg/mL kanamycin, and the plates were incubated at 37 °C for 24 h. Colony counts were then performed.

### 2.4. Data Analysis

All experimental data were analyzed using SPSS Statistics 26.0 (IBM Corp., Armonk, NY, USA). One-way analysis of variance (ANOVA) was used to compare chlorophyll content, plant height, root length, fresh weight, and Bt colony counts across treatments. Significant differences were identified using Duncan’s multiple range test at a significance level of *p* < 0.05. F-values, degrees of freedom (df), and *p*-values are reported accordingly.

## 3. Results

### 3.1. Pest Reduction Rate

In the control plot, where only water was sprayed without any pesticide treatment, the pest population showed a continuous increase over the 7-day post-treatment period, indicating a high natural growth rate of the pest population under environmental conditions and highlighting the ineffectiveness of water application in suppressing pest density. In contrast, the plots treated with the chemical pesticides PB-WP (Zu Jia^®^) and PB-ME (Jie Tiao^®^) exhibited significant pest suppression, with reduction rates of 54% and 50%, respectively, observed at one day post-treatment. This rapid decrease is consistent with the high efficacy typically seen in chemical pesticides due to their quick-acting insecticidal properties. However, the reduction rates declined progressively at 3 and 7 days post-treatment, demonstrating the tendency of these pesticides to lose effectiveness over time, which reflects the typical behavior of the specific chemical pesticide formulations used in this study, characterized by rapid onset but relatively limited residual activity under field conditions (F = 16.59; df = 1,10; *p* < 0.05) (F = 9.18; df =1,10; *p* < 0.05). Furthermore, this decline pattern may be influenced by factors such as the degradation rate, volatility of the chemical compounds under natural conditions, and environmental factors, thereby underscoring the limited persistence of chemical pesticides on plant surfaces.

**Figure 1:**
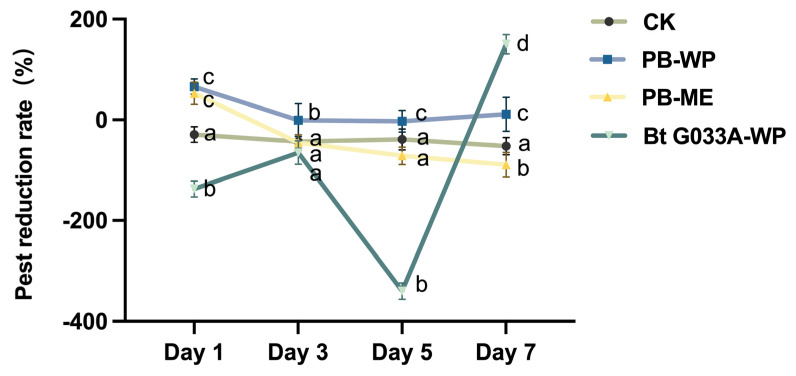
Efficacy of three pesticide treatments in reducing *P. striolata* population on *B. chinensis* over different time periods. Different letters above the bars indicate statistical significance (*p* < 0.05). Positive values indicate pest population reduction relative to initial numbers, while negative values indicate an increase in pest population.

In contrast, the treatment group using Bt G033A-WP exhibited a distinct pest suppression pattern. At one day post-treatment, pest density did not decrease; rather, it showed a slight increase, indicating the low immediacy of Bt’s efficacy. By day 3, the growth rate of pest populations slowed, and by day 7, a substantial reduction in pest density was observed, aligning with the characteristic slow-release effect typical of biological pesticides (F = 375.18; df = 1,10; *p* < 0.05). The delayed onset of efficacy may be attributed to Bt’s unique mechanism of action, wherein Bt spores must first adhere to and establish on the plant surface and then be ingested by *P. striolata*, undergo digestion, release toxic proteins, and subsequently impact the insect’s gut and cause mortality. This process inherently involves a latency period. Additionally, Bt formulations are highly sensitive to environmental factors such as sunlight, temperature, and precipitation, which can significantly impact Bt’s stability and colonization efficacy on plant surfaces. Under high temperatures, intense sunlight, or rainfall, Bt spore quantities may decrease, thereby extending the time required to achieve effective pest control (Figure 1).

### 3.2. Chlorophyll Content

Seven days after pesticide application, the chlorophyll content of healthy *B. chinensis* plants in each treatment plot was measured. The results showed that compared with the control group, the application of the chemical pesticides PB-WP (Zu Jia^®^) and PB-ME (Jie Tiao^®^) significantly reduced the chlorophyll content (F = 45.48; df = 1, 10; *p* < 0.0001 and F = 63.23; df = 1, 10; *p* < 0.0001, respectively). In contrast, the biopesticide Bt G033A-WP showed no significant difference in chlorophyll content compared with the control group (F = 1.91; df = 1, 10; *p* > 0.1).

The observed reduction in chlorophyll content caused by chemical pesticides may be attributed to their potential interference with plant metabolism. Previous studies suggest that certain chemical pesticides, while effectively controlling pests, may impact photosynthetic systems or pigment synthesis pathways directly or indirectly. Such effects could result from pesticide residues on the leaf surface or induced physiological stress responses, leading to pigment degradation or reduced photosynthetic efficiency. In contrast, Bt pesticides primarily target pests and generally do not interfere with plant physiological processes, which explains the nonsignificant effects on chlorophyll content.

These findings indicate that while chemical pesticide products are effective in controlling *P. striolata*, they may suppress chlorophyll synthesis in *B. chinensis*, raising concerns about potential impacts on non-target physiological parameters. Conversely, Bt pesticides demonstrated high ecological compatibility by effectively controlling pest populations without significantly affecting chlorophyll content.

**Figure 2:**
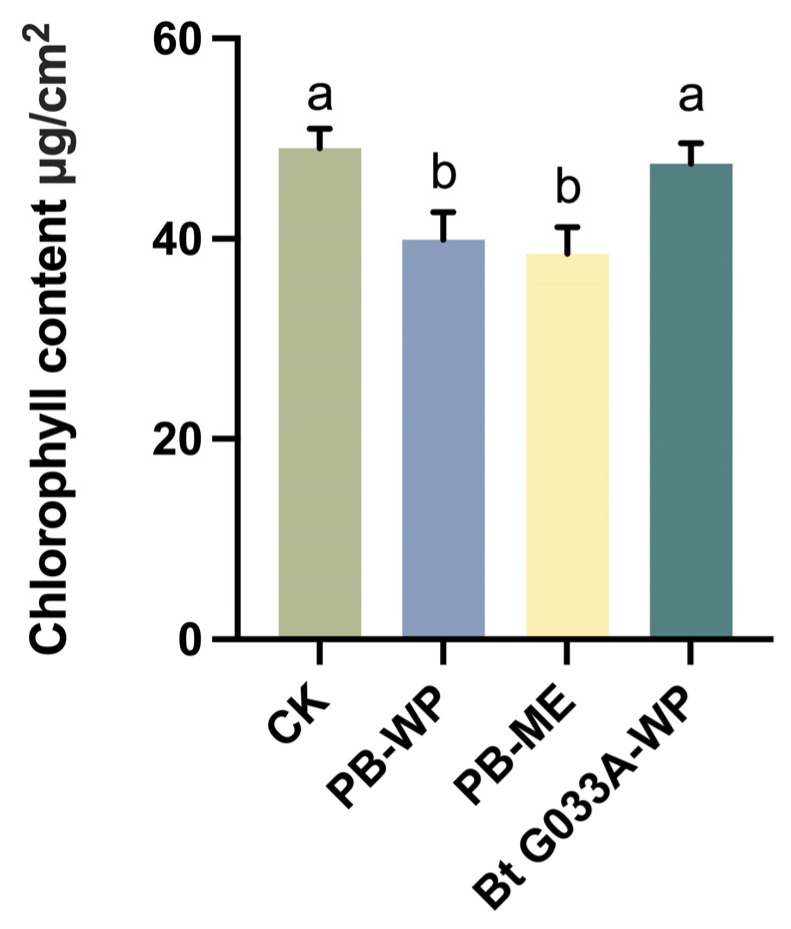
Effects of three pesticides on chlorophyll content in *B. chinensis*. Different letters above the bars indicate statistical significance (*p* < 0.05).

Overall, this study highlights the importance of balancing pest control efficacy with potential impacts on crop health when developing integrated pest management (IPM) strategies. The advantages of microbial pesticides like Bt, particularly in preserving photosynthetic capacity and crop metabolic stability, offer strong support for optimizing green control measures and reducing dependence on chemical pesticides (Figure 2).

### 3.3. Plant Growth Indicators

On the 7th day after pesticide application, the plant height and root length of healthy *B. chinensis* plants in each treatment plot were measured. The results showed that the plant height of the treatment groups sprayed with PB-WP (Zu Jia^®^) and PB-ME (Jie Tiao^®^) was significantly lower than that of the control group (F = 30.38; df = 1, 10; *p* < 0.01 and F = 37.93; df = 1, 10; *p* < 0.001, respectively). In contrast, the plant height of the group treated with Bt G033A-WP showed no significant differences from the control group (F = 0.0278; df = 1, 10; *p* > 0.05). Moreover, the root length in all treatment groups, whether chemical or biological pesticides, did not differ significantly from that of the control group (PB-WP: F = 0.3342; df = 1, 10; *p* > 0.05; PB-ME: F = 0.84; df = 1, 10; *p* > 0.05; Bt G033A-WP: F = 1.039; df = 1, 10; *p* > 0.05).

These findings suggest that the chemical pesticide treatments, specifically Zu Jia^®^ and Jie Tiao^®^, may adversely affect the aboveground growth (e.g., plant height) of *B. chinensis*, whereas biopesticide Bt exhibited no significant inhibitory effect on either plant height or root length. This differential response could reflect the potential impact of chemical pesticides on photosynthetic efficiency or cell elongation mechanisms in plants. In contrast, the specificity of the biological pesticide’s mode of action targeting insect digestive systems likely avoids interference with plant growth processes.

The differences in plant height responses underscore the need for cautious consideration of non-target physiological effects when using chemical pesticides, despite their efficacy in pest control. In comparison, Bt pesticides demonstrated minimal impact on plant morphological parameters while maintaining effective pest suppression, highlighting their superior safety profile for plant growth.

**Figure 3:**
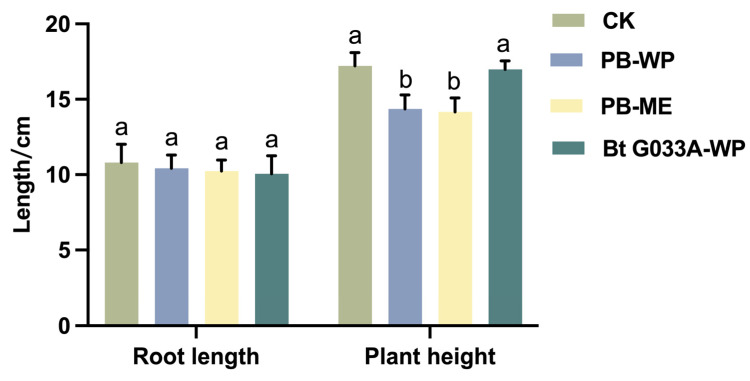
Effects of three pesticides on growth indicators of *B. chinensis*. Different letters above the bars indicate statistical significance (*p* < 0.05).

The relative stability of root length across all treatments indicates that, under the conditions of this study, the tested pesticides had limited effects on the underground growth of *B. chinensis*. This stability further supports the value of biological pesticides as a green pest control strategy in agricultural production. At the same time, the findings emphasize the importance of optimizing the dosage and application frequency of chemical pesticides to minimize their potential adverse effects on crop aboveground growth. In conclusion, this study demonstrates that while chemical pesticides effectively control pests, they may negatively affect the plant height of *B. chinensis*. Conversely, Bt not only achieves effective pest control but also maintains a high degree of safety for plant growth parameters. These findings provide valuable data to support the optimization of green pest control strategies in agriculture (Figure 3).

### 3.4. Plant Weight

On the 7th day after pesticide application, the fresh weight of healthy *B. chinensis* plants was measured to evaluate the impact of different pesticide treatments on plant growth. The results indicated that the application of the chemical pesticides PB-WP (Zu Jia^®^) and PB-ME (Jie Tiao^®^) significantly reduced the fresh weight of *B. chinensis* (F = 929.5; df = 1, 10; *p* < 0.05 and F = 196.99; df = 1, 10; *p* < 0.05, respectively). In contrast, the fresh weight of the group treated with the biological pesticide Bt G033A-WP was not significantly different from the control group (F = 1.575; df = 1, 10; *p* > 0.05).

The reduction in fresh weight observed in the chemical pesticide treatment groups suggests that these pesticides may inhibit biomass accumulation under the conditions tested. This inhibitory effect could be attributed to phytotoxicity or indirect physiological stress induced by the pesticides. Conversely, the Bt treatment group showed no significant inhibitory effects, highlighting its low phytotoxicity and high ecological compatibility while effectively controlling pests.

**Figure 4:**
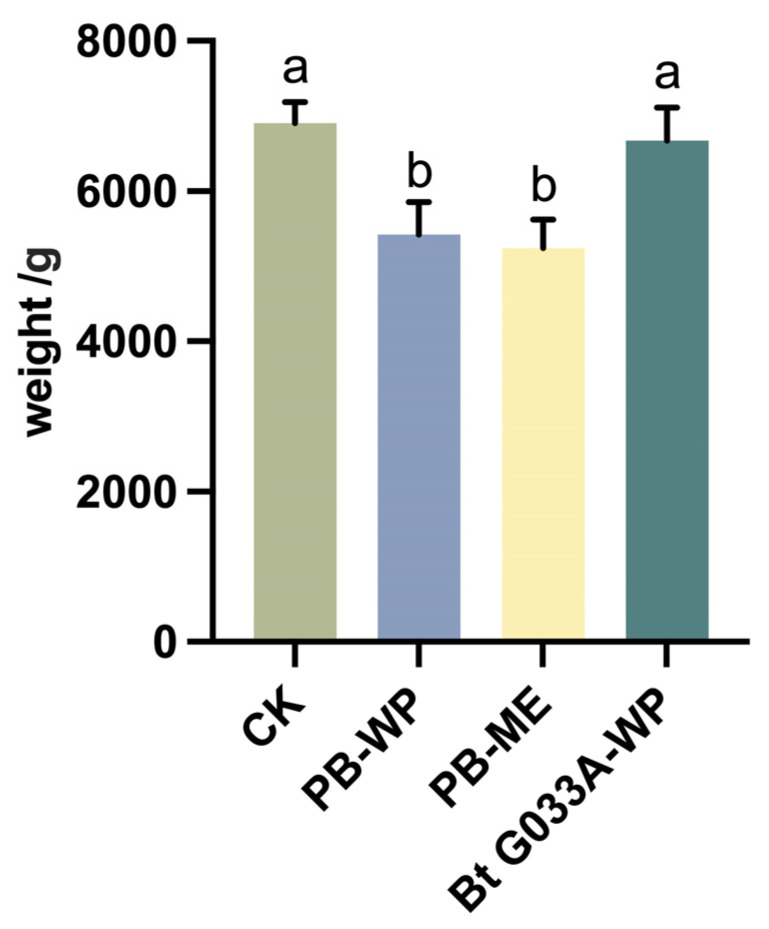
Effects of three pesticides on the weight of *B. chinensis*. Different letters above the bars indicate statistical significance (*p* < 0.05).

These findings underscore the importance of selecting appropriate pesticide types and application methods in pest control strategies. Compared to chemical pesticides, biological pesticides such as Bt have potential advantages in supporting crop growth and development, as well as in promoting sustainable agricultural management. This study provides valuable evidence for optimizing pesticide application strategies and emphasizes the importance of reducing chemical pesticide use. Biological pesticides, as environmentally friendly alternatives, demonstrate significant value in enhancing crop productivity and minimizing ecological impacts. Although nonsignificant, these trends warrant further investigation under larger-scale trials to assess practical agronomic implications. However, we acknowledge that the biological relevance of such trends remains uncertain at the field scale, and further validation under broader conditions is required (Figure 4).

### 3.5. Bacterial Colonization Count of Biopesticides

Leaf colony counts were performed on *B. chinensis* plants with vigorous growth from the group treated with the Bt G033A-WP to evaluate the colonization ability and persistence of Bt within the plant. The results showed that Bt effectively colonized the surface of *B. chinensis* leaves, forming stable colonies within seven days post-application. Compared to chemical pesticide treatments [PB-WP (Zu Jia^®^) and PB-ME (Jie Tiao^®^)], the colonization characteristics of Bt may provide a “natural barrier” during plant growth, offering sustained suppression of pest activity without significantly affecting plant growth parameters such as fresh weight. This observation suggests that, in contrast to conventional chemical pesticides, the Bt strain not only provides eco-friendly pest control but also may afford prolonged biological protection through persistent colonization within plant tissues.

**Figure 5:**
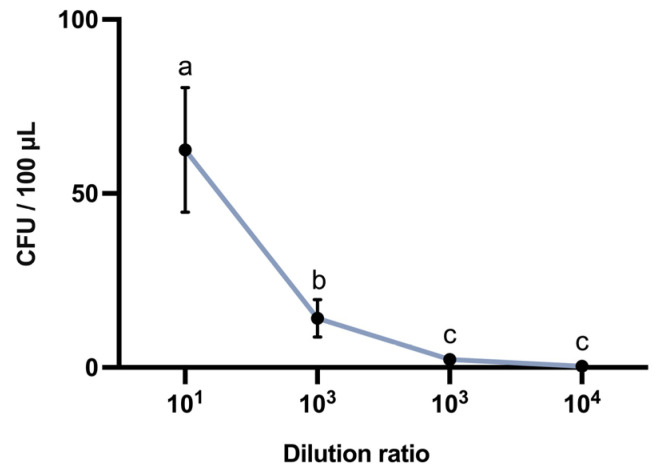
Bacterial colonization count on leaves after treatment with Bt G033A-WP. Different letters above the bars indicate statistical significance (*p* < 0.05).

Consequently, our findings highlight the potential application of microbial pesticides in sustainable agricultural management: the robust colonization of Bt strain G033A on crops can offer longer-lasting protection, while, unlike chemical agents, Bt application does not induce significant changes in plant fresh weight, indicating its low phytotoxicity and compatibility with ecological farming systems. Future studies may further explore the mechanisms of Bt colonization and its relationship with plant health to optimize strategies for biological pest control (Figure 5).

## 4. Discussion

This study comprehensively evaluated the control efficacy of the chemical pesticides PB-WP and PB-ME, as well as the microbial pesticide Bt G033A-WP. The results demonstrated that all three agents effectively controlled the striped flea beetle (*P. striolata*). However, compared to the control group, the chemical pesticides PB-WP and PB-ME significantly reduced the chlorophyll content, plant height, and fresh weight of *B. chinensis*. In contrast, the microbial pesticide Bt G033A-WP showed no significant impact on these growth parameters. These findings indicate that while chemical pesticides exhibit rapid action in pest management, their inhibitory effects on plant growth warrant attention. Meanwhile, Bt microbial pesticides, as a low-toxicity, environmentally friendly solution, demonstrated higher ecological compatibility, providing robust support for sustainable agriculture.

The application of the chemical pesticides PB-WP and PB-ME demonstrated high short-term efficacy in controlling striped flea beetles (*P. striolata*). However, their use significantly reduced the chlorophyll content, plant height, and fresh weight of *B. chinensis*, which may be attributed to the phytotoxic effects of these chemical pesticides [22,23,24,25]. Although the present data did not reveal significant effects on root length, prolonged use of these pesticides could have more profound negative impacts on crop metabolism and ecosystems, including harm to non-target organisms such as soil microbes and beneficial insects [26]. While the chlorophyll content analysis results provide insight into the immediate impact of chemical pesticides on plant photosynthesis, it is important to note that earlier studies have suggested that the phytotoxic effects of these pesticides extend beyond chlorophyll reduction. These effects may involve disruption of other metabolic pathways and cellular processes, which could contribute to the overall decline in plant health and productivity [27]. Future research should focus on the field degradation behavior of chemical pesticides, particularly their residue dynamics under different environmental conditions and their impacts on the microecological environment [28,29]. Such studies will be instrumental in optimizing the application strategies for chemical pesticides, such as reducing doses or application frequency, to strike a balance between effective pest management and environmental sustainability.

The biopesticide G033A-WP demonstrated excellent pest control efficacy in this study, without significant adverse effects on the chlorophyll content, plant height, root length, or fresh weight of *B. chinensis*. This indicates that Bt not only exerts its insecticidal effects on *P. striolata* through its toxin but may also form a stable microbial colony on plant surfaces, creating a sustained biological protective barrier [30]. We recognize that factors such as varying pest pressure across experimental plots and crop stress conditions could influence the outcomes of this study. While efforts were made to minimize these variables through randomization and replication, we acknowledge that these factors were not fully controlled for, and they could have contributed to some variability in the results. Future studies should aim to better control for these factors to provide more accurate and consistent data. While the slower onset of Bt’s effect has been attributed to colonization, we acknowledge that the evidence for its persistence under field conditions was not directly assessed in this study. This is an important area for future research, and further studies are needed to confirm the persistence of Bt colonies in real-world environments and their long-term efficacy. We also recognize that non-target effects and the practical challenges of scaling Bt production and application under various agricultural conditions were not fully addressed. Future research should investigate potential non-target impacts and explore methods to optimize Bt application in different agricultural systems. The prolonged efficacy of Bt may be attributed to the spore structure’s resistance to ultraviolet radiation and temperature fluctuations, making it a highly advantageous green pesticide [31]. However, it is important to acknowledge that environmental factors such as UV radiation intensity, temperature variability, and rainfall patterns can influence the survival and efficacy of Bt formulations in field conditions [32]. Although Bt spores are inherently more resistant to UV degradation compared to vegetative cells, prolonged exposure to strong UV radiation may still reduce their persistence and insecticidal activity over time [33]. Future research should explore formulation improvements (e.g., UV-protective additives or microencapsulation) to enhance field stability, as well as investigate the colonization mechanisms of Bt on crop surfaces and its interactions with other microorganisms to ensure its long-term efficacy across diverse cropping systems. However, this colonization was assessed through surface wash assays, which detect epiphytic Bt spores but do not allow differentiation between superficial presence and potential endophytic colonization within plant tissues. This represents a methodological limitation of our study. Future experiments should incorporate tissue maceration or molecular methods to determine whether Bt can persist internally as true endophytes. Further studies are needed to investigate whether Bt formulations can persist internally as true endophytes.

This study also demonstrated that the pest control efficacy of biopesticide G033A-WP is comparable to that of the chemical pesticides PB-WP and PB-ME against *P. striolata* but with significantly different impacts on plant growth. The environmentally friendly characteristics of Bt make it a promising alternative to chemical pesticides, effectively reducing the use of chemical inputs and their potential environmental impacts [34,35,36]. Although PB-WP and PB-ME offer rapid pest control, their significant inhibitory effects on plant chlorophyll content, height, and fresh weight underscore the need for more cautious application in future pest management strategies. A synergistic approach combining Bt and low-dose chemical pesticides could be an optimized solution: chemical pesticides can quickly reduce pest density, creating favorable conditions for Bt colonization, while Bt provides prolonged pest control efficacy. While promising, the synergistic effect of Bt combined with reduced-dose chemical pesticides remains hypothetical and should be directly tested in future field trials. As this study did not experimentally include such combinations, the actual operational relevance and synergistic potential of this approach in real-world IPM systems remain to be validated.

In summary, this study highlights the significant efficacy of the biopesticide G033A-WP in controlling *P. striolata* and its advantage of not adversely affecting plant growth. Although the chemical pesticides PB-WP and PB-ME demonstrated rapid control efficacy, their inhibitory effects on plant growth characteristics cannot be overlooked. A synergistic application of Bt and low-dose chemical pesticides could mitigate the negative impacts of chemical pesticides on plants and ecosystems, delay the development of pest resistance, and enhance overall pest control efficacy. Synergy is a hypothesis requiring further validation, and this has been proposed as a future research direction. Future research should focus on determining the optimal ratio and application timing for Bt and low-dose chemical pesticides, as well as their long-term efficacy under field conditions. The experimental timeline used in this study is sufficient for evaluating short-term efficacy, but further studies with extended timeframes are necessary to fully assess pest resistance dynamics and potential long-term ecosystem impacts. Nonetheless, the 7-day observation period still restricts the evaluation of long-term residual activity and potential pest population rebound under real field conditions. Future research should extend the observation period to assess the long-term persistence of pest suppression and the potential for pest population rebound under variable field conditions. Additionally, attention should be paid to potential impacts on non-target organisms and ecosystems to ensure the safety and effectiveness of this synergistic approach. This study provides scientific evidence for developing efficient and environmentally friendly integrated pest management (IPM) strategies, contributing to the sustainable development goals of agricultural production. While this study showcased the rapid degradation of chemical pesticides and the comparatively safer, albeit slower, action of Bt-based biopesticide, its long-term applicability to integrated pest management (IPM) remains questionable. The experiment was carried out over a limited observation period of three weeks and did not include tests on the combined use of Bt and chemical pesticides. Future field trials should feature longer observation periods and combined applications of Bt and chemical pesticides. This will allow for a comprehensive evaluation of their potential synergy and practical viability for the sustainable management of the *P. striolata*.

## 5. Conclusions

This study demonstrates that biopesticides are effective in controlling *P. striolata* populations and promoting the growth of *B. chinensis*, with no adverse effects on chlorophyll content. Compared to two chemical pesticides, biopesticides showed promising results in reducing pest density and improving plant growth, while offering higher environmental sustainability. However, chemical pesticides may provide faster pest control, particularly under high pest pressure. Therefore, integrating the advantages of both biopesticides and chemical pesticides into an integrated pest management strategy may represent a more sustainable and efficient approach for future agricultural production.

## Figures and Tables

**Table 1:** The active ingredients, formulations, concentrations, and abbreviations of the pesticides used in the experiment.

Trade Name	Active Ingredient	Formulation	Concentration	Abbreviations Used in this Paper
Zu Jia^®^	21% Imidacloprid 21% Abamectin	Wettable powder	750 g/ha in 1 g/L water	PB-WP
Jie Tiao^®^	10% Imidacloprid 10% Abamectin	Microemulsion	375 mL/ha in water	PB-ME
*Bacillus thuringiensis* G033A	32,000 IU/mg *Bacillus thuringiensis* (strain G033A)	Wettable powder	1.3 kg/ha in 1 g/L water	Bt G033A-WP

Note: All application rates were fixed according to the manufacturers’ recommendation and applied uniformly across plots. No variation in concentration or rate was observed.

## Data Availability

The original contributions presented in this study are included in the article. Further inquiries can be directed to the corresponding author.
